# MicroCT evaluation for CAD/CAM occlusal veneer fit using two materials and three cement space settings

**DOI:** 10.1590/0103-6440202204764

**Published:** 2022-08-26

**Authors:** Adel Abdelsattar Elbadawy, Elsayed Ali Omar, Mohammed Hosny AbdElaziz

**Affiliations:** 1 Department of Crown and Bridge, Faculty of Dental Medicine, Al Azhar University, Cairo, Egypt; 2 Department of Prosthodontics, College of dentistry, Taif University. Al Hawiyah, Saudi Arabia; 3 Department of Substitutive Dental Sciences, Taibah University. Madinah, Saudi Arabia

**Keywords:** Occlusal veneer, marginal and internal gaps, cement space, microCT evaluation, CAD/CAM

## Abstract

This study was aimed to evaluate the fit of occlusal veneer restoration for two CAD/CAM materials with different cement space settings, using microCT scans. Sixty resin dies were made and divided into two groups (n=30) according to the materials, (I): Hybrid all-ceramic, and (II): zirconia-reinforced lithium silicate glass-ceramic. Each group was subdivided into three subgroups (n=10) according to the cement space parameters (30, 40, and 50 µm). Occlusal veneers for the six subgroups were milled. A circle with 20 different sections was placed at the center of every scanned specimen to measure four different locations (Occlusal, Axial, Marginal, and Absolute marginal discrepancy). Data were analyzed using two-way ANOVA at a 0.05 level of significance. There was no statistically significant effect of material type on the mean values of internal and marginal gaps for the three cement space parameters (P>0.05). There were no statistically significant differences in the occlusal and axial gap between the cement space parameters, furthermore, there were statistically significant differences in marginal gap distances and absolute marginal discrepancies (P>0.05). Hybrid all-ceramic showed smaller marginal and internal discrepancies than zirconia-reinforced lithium silicate glass-ceramic without statistically significant differences, and, for both materials, 50 µm cement space significantly improved the marginal fit and absolute marginal discrepancy.

## Resumo

Este estudo teve como objetivo avaliar o ajuste da restauração de facetas oclusais para dois materiais CAD/CAM com diferentes configurações de espaço de cimento, usando microCT. Foram feitos sessenta moldes de resina e divididos em dois grupos (n=30) de acordo com os materiais, (I): cerâmica pura híbrida, e (II): cerâmica de vidro de silicato de lítio reforçada com zircónia. Cada grupo foi subdividido em três subgrupos (n=10), de acordo com os parâmetros do espaço de cimento (30, 40, e 50 µm). As facetas oclusais para os seis subgrupos foram fresadas. Um círculo com 20 secções diferentes foi colocado no centro de cada amostra digitalizada para medir quatro locais diferentes (Oclusal, Axial, Marginal, e discrepância marginal absoluta). Os dados foram analisados utilizando ANOVA bidireccional a um nível de significância de 0,05. Não houve efeito estatisticamente significativo do tipo de material nos valores médios das aberturas internas e marginais para os três parâmetros do espaço de cimento (P>0,05). Não houve diferenças estatisticamente significativas no intervalo oclusal e axial entre os parâmetros do espaço de cimento, além disso, houve diferenças estatisticamente significativas nas distâncias do intervalo marginal e discrepâncias marginais absolutas (P>0,05). A cerâmica pura híbrida mostrou discrepâncias marginais e internas menores do que a cerâmica de vidro de silicato de lítio reforçada com zircónia sem diferenças estatisticamente significativas, e, para ambos os materiais, 50 µm de espaço de cimento melhoraram significativamente o ajuste marginal e a discrepância marginal absoluta.

## Introduction

Extensive destruction of occlusal enamel leads to loss of occlusal contacts and ultimately to the formation of parafunctional activities ^1^. The use of a full-coverage crown could be considered a debatable intervention as it involves additional loss of tooth structures for the already affected dentition ^2^. For this reason, occlusal veneer restoration represents an appropriate treatment modality ^3^ ^,^ ^4^ ^,^ ^5^. Improved CAD-CAM technologies and materials, contributed to the introduction of precise restorations with a reasonable fit for non-retentive conservative preparations with a minimal thickness which still enjoys excellent esthetic and mechanical properties ^6^.

Recently, hybrid ceramic material has been presented, characterized by its high degree of strength and elasticity, therefore, allowing to use of thinner restorations ^7^ ^,^ ^8^ ^,^ ^9^ ^,^ ^10^. Optimization of Lithium silicate glass-ceramic with approximately 10 wt % zirconia provides positive characteristics with improved translucency. It stands out as a new aesthetic, strong, and applicable material for dental CAD/CAM restorations ^11^. In addition to preparation design, the impression technique, and the milling procedure, the digital cement space setting plays an important role in the marginal and internal accuracy of the CAD/CAM restorations ^12^. Poor marginal adaptation can affect the integrity of the cement seal leading to caries, plaque accumulation, and finally, periodontal diseases. In addition, an increase in the internal gap could further decrease the fracture strength of ceramic restorations due to the different load concentrations in these areas ^13^.

Die spacers application has been effectively used for heat-press and lost-wax procedures to produce suitable marginal and internal gaps, allowing crown restorations to be fully seated. Crown restorations created with (CAD/ CAM) technologies, on the other hand, normally have their spacer thickness specified during the software's design phase. However, the most appropriate spacer thickness setting for proper occlusal veneer adaptation is uncertain ^14^. To assess the marginal and internal adaptation, sample sectioning followed by microscopic evaluation was used, but this method is a destructive procedure in addition to the loss of some details of the samples ^15^ ^,^ ^16^. The microcomputed tomography (microCT) scan is considered the most efficient and non-destructive investigational approach for both marginal and internal adaptations ^17^.

This study was directed to assess the marginal and internal adaptation for occlusal veneer restoration using two types of CAD/CAM materials (zirconia-reinforced lithium silicate glass-ceramic and polymer-infiltrated ceramic network) with three different cement space settings (30 µm, 40 µm, and 50 µm), using microCT scans. The null hypotheses to be tested were that there are no differences in the marginal and internal adaptations between ^1^ the two materials used to fabricate the occlusal veneer and ^2^ the three tested digital cement space parameters.

## Materials and methods

### Tooth preparation

**Figure. 1:**
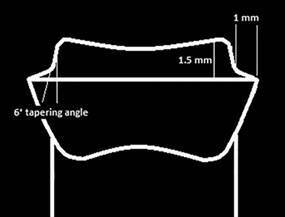
Schematic representation for Occlusal veneer preparation guidelines.

A typodont mandibular right first molar (KaVo Dental, Biberach, Germany) was prepared for occlusal veneers by a medium grit tapered diamond bur with a rounded end, using a milling machine (BEGO. PARASKOP, Bremen, Germany). The tooth was prepared as follows: 1.5 mm height extending from the margin to the occlusal plane, 1 mm circumferential rounded chamfer, and rounded Axio-gingival angle with 1.0 mm curvature radius ^18^. 1.5 mm axial reduction with 6° tapering angle, 1.0-1.5 mm anatomically shaped occlusal reduction ^19^. (Figure 1) Finally, three dimples were made at the level of cementoenamel junction on mesial, distal, and buccal surfaces using round diamond bur) they were used as guides to standardize the position of the die and its corresponding occlusal veneer within the mold during scanning).

### Master die replication and sample grouping

60 resin master dies were made by replicating the prepared tooth using the Cerec inLab 3D system (Sirona, GmbH. D-64625 Bensheiu, German). The dies were randomly and equally assigned to two groups (n=30) according to the materials, group (I): zirconia-reinforced lithium silicate glass-ceramic (Vita Suprinity; VITA Zahnfabrik, Bad Säckingen, Germany), and group (II): Hybrid all-ceramic material (Vita Enamic, VITA Zahnfabrik, Bad Säckingen, Germany) (Table 1). Then, each group was subdivided into three equal subgroups (n=10) according to the cement space parameters (30, 40, and 50 µm) ^12^ ^,^ ^21^.

**Table 1:** Brands, abbreviations, description, composition, and manufacturers of the materials used in this study.

Brand name	Description	Composition	Manufacturer
Vita Suprinity (VS)	zirconia-reinforced lithium silicate glass - ceramic	10% zirconia glass - ceramic	VITA Zahnfabrik
Vita Enamic (VE)	polymer-infiltrated ceramic network	86% fine - structure feldspar ceramic,14% polymer	VITA Zahnfabrik

### Digital impression, designing, milling, and crystallization

The master dies were homogenously sprayed using fluorinated hydrocarbon pigment suspension (Optispray, Sirona Dental Systems GmbH), then digital impressions of all master dies were made using the Cerec scanner (Ineos Blue scanner, Sirona Dental Systems GmbH). The same occlusal veneer design with identical external contours for all groups was made. A simulated cement space of 25 µm strap of 0.5 µm above the finish lines was designed, then, additional cement spaces of 30 µm, 40 µm, and 50 µm were set forming the following three tested subgroups for each type of material, a (25-30), B (25-40), and C (25-50) ^19^ ^,^ ^20^ ^,^ ^21^ ^,^ ^22^.

After designing each type of occlusal veneer material, the data was sent to the Cerec in Lab milling unit (Sirona dental system GmbH. D-64625 Bensheim, German) for occlusal veneers milling. After the milling process, occlusal veneers for the vita suprinity samples were crystallized in program at the P310 furnace (Programat P310, Ivoclar Vivadent AG, Schaan/Liechtenstein). No adjustments were made to the ceramic veneers before marginal and internal adaptation measurements ^18^ ^,^ ^23^.

### Sample’s position standardization

To ensure standardization of the sample’s position during scanning, one master dies with its corresponding occlusal veneer placed in a mold with dimensions of 4X3X3 cm filled with light body polyvinyl siloxane (3M, ESPE, Express, Dental Products, St Paul, USA) from its buccal side until mesial and distal dimples are covered, then, a stainless steel wire (0.1mm) was embedded parallel to the longitudinal axis of the mesial side (used as a fixed starting reference point) ^19^.

### MicroCT Scan procedures

**Figure 2:**
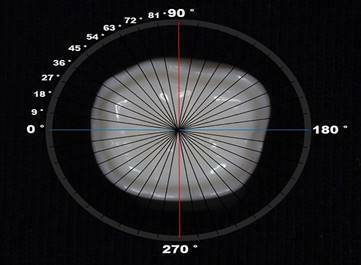
Schematic representation for samples segmentation on the resin die.

The occlusal veneers were seated onto their corresponding dies without any luting medium ^20^. A circle with 20 different sections (wa ith 9° increase for each) was placed at the center of the standardized position of every scanned specimen ^19^ ^,^ ^24^ as illustrated in (Figures 2 and 3) to measure four different locations as follow: -

[1] Occlusal gap: the mean of the three occlusal values: After dividing the length of the occlusal surface into four equal parts, one measurement at the middle and one at the middle of each half were taken.

[2] Axial gap: The mean of two right and left axial values: in the middle of axial walls.

[3] Marginal gap: The mean of two right and left marginal values at the middle of the finish line.

[4] Absolute marginal discrepancy value (AMD): The mean of two right and left distances between the margin of occlusal veneer and die margin ^12^ ^,^ ^16^.

Images with a resolution of 9.77 μm /pixel were acquired from 20 slices and were used to measure four sites per slice (occlusal, axial, marginal, and absolute marginal discrepancy) with a total of 80 measuring values for each tooth ^12^ ^,^ ^22^. Each tooth was scanned using microCT (Viscom X8060, Viscom AG, Hannover, Germany) with the following setting parameters: Operation at 130 kV, tube current at 160 mA, a 5 - mm thick aluminum plate, 15 X magnification, 4.9 s exposure time.

**Figuere 3:**
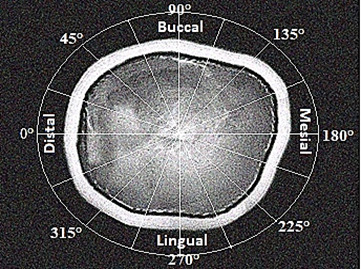
2D schematic representation for samples segmentation on the horizontal CT scan image.

### Measurement procedures

The digital data were developed using reconstruction software (XVRCT, Viscom AG, software version 1.07), and converted into a tagged image file format (TIFF) for the subsequent segmentation (Definiens Developer XD 2.1.1, Definiens AG, Munich, Germany). Using (trabecular thickness) algorithm option by the image processing and analyzing software (Image J, FUJI); the absolute marginal discrepancy (AMD), marginal and internal adaptation have been measured (^25^ ^,^ ^26^ ^,^ ^27^.

### Statistical analysis

The sample size and power analysis were calculated using statistical software (nQuery Advisor, version 7.0). The sample mean and standard deviation variance was 84.19 ± 13.69; accordingly, a sample size of 30 per group (10 per cement space parameter) was considered satisfactory to obtain a type I error for power more than 0.99 of alpha of 0.05.

Statistical analyses were performed with statistical software (IBM SPSS Statistics v22.0; IBM Corp). The Shapiro-Wilk test of normality confirmed that the data were normally distributed (P>.05).

A Two-way ANOVA test has been used to identify the main effect of material types and cement space settings on each site of measurement (Occlusal, Axial, Marginal and absolute marginal discrepancy) followed by One - way ANOVA with post hoc tests (LSD) for each effective variable (exhibited statistical significant difference) separately within cement space parameters.

## Results

A total of 1200 images were obtained for all tested samples (20 sections X 60 samples), including ten occlusal veneer /subgroup and four measuring value /slice with 4800 measuring values. A summary of mean gap values, standard deviations, minimum and maximum gap values) in micrometer (µm) for both materials (Vita Enamic and Vita Suprinity) at the four tested sites (Occlusal, Axial, Marginal, and Absolute marginal discrepancy) for the three tested cement spaces (30, 40, and 50 µm) are shown in (Table 2).

Regarding the effect of material factor, the two-way ANOVA test indicated no statistically significant effect for the four tested locations (occlusal, axial, marginal, and absolute marginal discrepancy) (P -values = 0.121, 0.09, 0.233, and 0.168 respectively). (Table 3)

Regarding the effect of the cement spaces factor, the two-way ANOVA test indicated that there was no statistically significant effect for both occlusal and axial sites) (P-Value = 0.265 and 0.06 respectively) while there was a statistically significant effect for both marginal and absolute marginal discrepancy (P-Value = 0.00 for both). (Table 3)

**Table 2:** Descriptive means and standard deviations of internal and marginal gaps in micrometer (µm) for both materials at the four tested sites.

		Occlusal	Axial	Marginal	AMD
Vita Enamic	30 µm	90.41±8.67	80.15±6.96	75.47±6.97	96.04±4.88
	40 µm	91.33±5.43	83.55±5.68	69.24±7.04	90.22±5.93
	50 µm	94.19±8.18	84.42±6.83	56.34±7.67	79.88±8.36
Vita Suprinity	30 µm	92.37±9.92	82.60±5.88	77.06±6.31	99.35±9.25
	40 µm	96.46±9.81	86.19±8.29	72.53±7.06	93.42±5.64
	50 µm	97.23±7.84	87.93±5.23	57.79±6.17	80.76±6.45

AMD: absolute marginal discrepancy

**Table 3:** Two - way ANOVA results for the main effect of materials and cement spaces factors

Location	Source	Sum of Squares	df	Mean Square	F Value	P- Value
Occlusal	Materials	171.366	1	171.366	2.478	.121
	Spaces	188.361	2	94.181	1.362	.265
Axial	Materials	123.267	1	123.267	2.969	.090
	Spaces	246.207	2	123.103	2.965	.060
Marginal	Materials	66.908	1	66.908	1.456	.233
	Spaces	3923.826	2	1961.913	42.696	.000*
AMD	Materials	90.996	1	90.996	1.952	.168
	Spaces	3122.798	2	1561.399	33.502	.000*

* P Value is significant at the 0.05 level. AMD: absolute marginal discrepancy

One-way ANOVA test for multiple comparison statistics at marginal and absolute marginal discrepancy revealed that from 30 to 40 µm cement spaces, there were no statistically significant differences in the gap distances for both tested materials (P values = 0.064 and 0.057 respectively for Vita Enamic) (P-values = 0.132 and 0.08 respectively for Vita Suprinity). From 30 to 50 µm and from 40 to 50 µm there were statistically significant differences in the gap distances for both tested materials (P values = 0.000). (Table 4 and Table 5)

**Table 4:** Multiple comparisons statistical results for cement spaces at the marginal gap and absolute marginal discrepancy for Vita Enamic.

		Spaces µm	Mean Difference	Std. Error	P- Value	95% Confidence Interval	
						Lower Bound	Upper Bound
Vita Enamic	Marginal	30 -- 40	6.239	3.235	.064	3989	12.877
		30 -- 50	19.134^*^	3.235	.000	12.496	25.772
		40 -- 50	12.895^*^	3.235	.000	6.257	19.533
	AMD	30 -- 40	5.822	2.932	.057	1946	11.839
		30 -- 50	16.157^*^	2.932	.000	10.140	22.174
		40 -- 50	10.335^*^	2.932	.002	4.318	16.352

*. The mean difference is significant at the 0.05 level. AMD: absolute marginal discrepancy

**Table 5:** Multiple comparisons statistical results for cement spaces at the marginal gap and absolute marginal discrepancy for Vita Suprinity.

		Spaces µm	Mean Difference	Std. Error	P- Value	95% Confidence Interval	
						Lower Bound	Upper Bound
Vita Suprinity	Marginal	30 -- 40	4.537	2.919	.132	1.451	10.525
		30 -- 50	19.270^*^	2.919	.000	13.282	25.258
		40 -- 50	14.733^*^	2.919	.000	8.745	20.721
	AMD	30 -- 40	5.929	3.255	.080	.750	12.608
		30 -- 50	18.585^*^	3.255	.000	11.910	25.264
		40 -- 50	12.656^*^	3.255	.001	5.977	19.335

*. The mean difference is significant at the 0.05 level. AMD: absolute marginal discrepancy

## Discussion

The clinical significance of this study is that the outcomes may help in the clinical assessment of the least cement space setting providing optimal marginal adaptation for Hybrid all-ceramic, and zirconia-reinforced lithium silicate glass-ceramic occlusal veneers.

Based on the results of this study; cement space parameters only affected the marginal adaptation of occlusal veneer restorations, while, the type of material did not affect, so the null hypothesis would be partially rejected.

The material’s relationship with the luting agents plays an important role in the clinical success of occlusal veneer restorations ^28^ ^,^ ^29^, therefore, the selection of the materials that were used in this study (polymer-infiltrated ceramic network and Zirconia reinforced lithium silicate) based on their capacity to be etched and bonded to the adhesives ^30^.

A previous study has postulated that the ideal value for marginal adaptation should be 100 to 200 µm to be clinically acceptable ^31^. Another study has considered that it should be less than 100 µm. ^15^ Therefore, the results of marginal and internal gaps for all tested groups presented in this study can be considered clinically acceptable. The marginal gap values for this study were lower than those reported by other studies ^16^ ^,^ ^20^. These differences may be attributed to the differences in scanning accuracy, used materials, or preparation designs.

In this study, an extremely high resolution of microCT (9.77 μm) was used. The high resolution increases the contrast of the image layers, which subsequently improves the gap measurements. The microscopic evaluation inherently involves projection error due to the frontal view contrary to the sectional view used in microCT. Furthermore, the standard selection of measurement points around the multiple replicas is challenging ^32^.

In the present study, the same reproduced die was used for each veneer, and standardized positions were used for scanning. To ensure the standardization of the computed tomography images for all slices, each die with its corresponding occlusal veneer was placed in a prefabricated special mold with thin stainless-steel wire (0.1mm) parallel to the longitudinal axis of the mesial side as a fixed starting reference point.

Manual adjustment of restorations after visual inspection could greatly decrease the marginal gap and AMD, but this technique was not applied in this study contrary to other studies ^33^ ^,^ ^34^.

Polymer-infiltrated ceramic exhibited marginal and internal gap values lower than that for zirconia-reinforced lithium silicate ceramic without statistically significant differences, this may be attributed to reduced brittleness of polymer-infiltrated ceramic providing thin and smooth margin which enhances the milling procedure during manufacturing process unlike Zirconia reinforced lithium silicate, which retains their crystal structure and rupture during the milling process ^10^.

The mean internal gap values of all tested groups did not imitate the designed cement spaces in the design software, this result is in accordance with that of the other studies ^23^ ^,^ ^35^. This may be attributed to the presence of premature contacts within the fitting surface which may change the proper seating of the occlusal veneer and consequently increase the internal and marginal discrepancies ^20^.

In agreement with other previous studies ^20^ ^,^ ^23^ ^,^ ^36^, the results of the present study revealed an inverse relationship between marginal gap distance and cement space distance with statistically significant differences among the different virtual cement space settings within the same type of material as the spacer setting compensates for the inaccuracies of the fabrication workflow (which may interfere with restoration’s complete seating) to minimize the marginal gap. Therefore, a lower spacer setting than factory recommendation may inversely increase the gap ^32^.

The absolute marginal discrepancy is the distance between the preparation margin and restoration margin (It is the hypotenuse of a triangle whose sides are marginal gap and horizontal marginal overextension), therefore, it must be greater than or in an ideal state equal to the marginal gap ^37^. This study revealed that increasing cement space setting from 30 µm to 50 µm resulted in a lower absolute marginal discrepancy, this result is in agreement with other studies ^36^ ^,^ ^38^.

Absolute marginal discrepancy values of this study were lower than those evaluated by Yildirim et al using vita Enamic crowns ^23^. The explanation of this difference may be attributed to using a different type of restoration (occlusal veneers instead of full - coverage crowns)

Based on the microCT measurements, the comparison of the different virtual cement space settings for each material showed a statistically significant difference concerning the accuracy of fit. These results indicate that an increase in the cement space setting parameters improves the marginal fit. This result is in agreement with Shim et al ^36^.

In this study, axial mean gap values were lower than those at the occlusal surface. This is similar to other studies ^39^ ^,^ ^40^, the explanation of this difference may be attributed to that, that axial walls are milled with the side of the instrument bur. where the occlusal surface is milled with the tip of the bur.

The major disadvantage of microCT evaluation is the formation of radiation artifacts caused by the differences of radiation absorption coefficients between different materials during the evaluation of the gap distances ^41^, in addition to the presence of radiation artifacts, time consumption, a need for technical knowledge, and high cost ^42^. Based on the results of this study, the following conclusions were drawn: 1. Vita enamic groups showed smaller marginal and internal discrepancies than vita suprinity groups without statistically significant differences. 2. The marginal and internal gap values were within the clinically acceptable range, and 3. For both vita enamic and vita suprinity occlusal veneer, 50 µm cement space significantly improved the marginal fit and absolute marginal discrepancy but had no statistically significant effect on the internal gap values.

## References

[1] Schlichting LH, Resende TH, Reis KR, Magne P (2016). Simplified treatment of severe dental erosion with ultrathin
CAD-CAM composite occlusal veneers and anterior bilaminar
veneers. J Prosthet Dent.

[2] Varma S, Preiskel A, Bartlett D (2018). The management of tooth wear with crowns and indirect
restorations. British dental journal. Br Dent J.

[3] Guess PC, Schultheis S, Wolkewitz M, Zhang Y, Strub JR (2013). Influence of preparation design and ceramic thicknesses on
fracture resistance and failure modes of premolar partial coverage
restorations. J Prosthet Dent.

[4] Muts EJ, van Pelt H, Edelhoff D, Krejci I, Cune M (2014). Tooth wear: a systematic review of treatment
options. J Prosthet Dent.

[5] Ioannidis A, Bomze D, Hämmerle CHF, Hüsler J, Birrer O, Mühlemann S (2020). Load-bearing capacity of CAD/CAM 3D-printed zirconia, CAD/CAM
milled zirconia, and heat-pressed lithium disilicate ultra-thin occlusal
veneers on molars. Dent Mater.

[6] Magne P, Magne M, Belser UC (2007). Adhesive restorations, centric relation, and the Dahl principle:
minimally invasive approaches to localized anterior tooth
erosion. Eur J Esthet Dent.

[7] Kurbad A, Kurbad S (2013). A new, hybrid material for minimally invasive restorations in
clinical use. Int J Comput Dent.

[8] Awada A, Nathanson D (2015). Mechanical properties of resin-ceramic CAD/CAM restorative
materials. J Prosthet Dent.

[9] Mainjot AK, Dupont NM, Oudkerk JC, Dewael TY, Sadoun MJ (2016). From Artisanal to CAD-CAM Blocks: State of the Art of Indirect
Composites. J Dent Res.

[10] Furtado de Mendonca A, Shahmoradi M, Gouvêa CVD, De Souza GM, Ellakwa A (2019). Microstructural and Mechanical Characterization of CAD/CAM
Materials for Monolithic Dental Restorations. J Prosthodont.

[11] Curran P, Cattani-Lorente M, Anselm Wiskott HW, Durual S, Scherrer SS (2017). Grinding damage assessment for CAD-CAM restorative
materials. Dent Mater.

[12] Dauti R, Lilaj B, Heimel P, Moritz A, Schedle A, Cvikl B (2020). Influence of two different cement space settings and three
different cement types on the fit of polymer-infiltrated ceramic network
material crowns manufactured using a complete digital
workflow. Clin Oral Investig.

[13] Souza RO, Ozcan M, Pavanelli CA, Buso L, Lombardo GH, Michida SM (2012). Marginal and internal dicrepancies related to margin design of
ceramic crowns fabricated by a CAD/CAM system. J Prosthodont.

[14] Mously HA, Finkelman M, Zandparsa R, Hirayama H (2014). Marginal and internal adaptation of ceramic crown restorations
fabricated with CAD/CAM technology and the heat-press
technique. J Prosthet Dent.

[15] Nawafleh NA, Mack F, Evans J, Mackay J, Hatamleh MM (2013). Accuracy and reliability of methods to measure marginal
adaptation of crowns and FDPs: a literature review. J Prosthodont.

[16] Riccitiello F, Amato M, Leone R, Spagnuolo G, Sorrentino R (2018). In vitro Evaluation of the Marginal Fit and Internal Adaptation
of Zirconia and Lithium Disilicate Single Crowns: Micro-CT Comparison
Between Different Manufacturing Procedures. Open Dent J.

[17] Kim JH, Jeong JH, Lee JH, Cho HW (2016). Fit of lithium disilicate crowns fabricated from conventional and
digital impressions assessed with micro-CT. J Prosthet Dent.

[18] Neves FD, Prado CJ, Prudente MS, Carneiro TA, Zancopé K, Davi LR (2014). Micro-computed tomography evaluation of marginal fit of lithium
disilicate crowns fabricated by using chairside CAD/CAM systems or the
heat-pressing technique. J Prosthet Dent.

[19] Elbadawy AA, Elaziz MHA, Alnazzawi AA, Borzangy SS (2021). Effect of various digital cement space settings on the adaptation
of CAD/CAM occlusal veneer "micro-ct evaluation". Dent mater j.

[20] Kale E, Seker E, Yilmaz B, Özcelik TB (2016). Effect of cement space on the marginal fit of CAD-CAM-fabricated
monolithic zirconia crowns. J Prosthet Dent.

[21] Şeker E, Ozcelik TB, Rathi N, Yilmaz B (2016). Evaluation of marginal fit of CAD/CAM restorations fabricated
through cone beam computerized tomography and laboratory scanner
data. J Prosthet Dent.

[22] Özçelik TB, Yilmaz B, Şeker E, Shah K (2018). Marginal Adaptation of Provisional CAD/CAM Restorations
Fabricated Using Various Simulated Digital Cement Space
Settings. Int J Oral Maxillofac Implants.

[23] Yildirim G, Uzun IH, Keles A (2017). Evaluation of marginal and internal adaptation of hybrid and
nanoceramic systems with microcomputed tomography: An in vitro
study. J Prosthet Dent.

[24] Krasanaki ME, Pelekanos S, Andreiotelli M, Koutayas SO, Eliades G (2012). X-ray microtomographic evaluation of the influence of two
preparation types on marginal fit of CAD/CAM alumina copings: a pilot
study. Int J Prosthodont.

[25] Doube M, Kłosowski MM, Arganda-Carreras I, Cordelières FP, Dougherty RP, Jackson JS (2010). BoneJ: Free and extensible bone image analysis in
ImageJ. Bone.

[26] Schneider CA, Rasband WS, Eliceiri KW (2012). NIH Image to ImageJ: 25 years of image analysis. Nat Methods.

[27] Schindelin J, Arganda-Carreras I, Frise E, Kaynig V, Longair M, Pietzsch T (2012). Fiji: an open-source platform for biological-image
analysis. Nat Methods.

[28] Lima JM, Souza AC, Anami LC, Bottino MA, Melo RM, Souza RO (2013). Effects of thickness, processing technique, and cooling rate
protocol on the flexural strength of a bilayer ceramic
system. Dent Mater.

[29] Rekow ED, Silva NR, Coelho PG, Zhang Y, Guess P, Thompson VP (2011). Performance of dental ceramics: challenges for
improvements. J Dent Res.

[30] Santos MJ, Freitas MC, Azevedo LM, Santos GC, Navarro MF, Francischone CE (2016). Clinical evaluation of ceramic inlays and onlays fabricated with
two systems: 12-year follow-up. Clin Oral Investig.

[31] Renne W, McGill ST, Forshee KV, DeFee MR, Mennito AS (2012). Predicting marginal fit of CAD/CAM crowns based on the presence
or absence of common preparation errors. J Prosthet Dent.

[32] Vág J, Nagy Z, Bocklet C, Kiss T, Nagy Á, Simon B (2020). Marginal and internal fit of full ceramic crowns milled using
CADCAM systems on cadaver full arch scans. BMC oral health.

[33] Tabata LF, de Lima Silva TA, de Paula AC, Ribeiro APD (2020). Marginal and internal fit of CAD-CAM composite resin and ceramic
crowns before and after internal adjustment. J Prosthet Dent.

[34] Gressler May L, Kelly JR, Bottino MA, Hill T (2015). Influence of the resin cement thickness on the fatigue failure
loads of CAD/CAM feldspathic crowns. Dent Mater.

[35] Prudente MS, Davi LR, Nabbout KO, Prado CJ, Pereira LM, Zancopé K (2018). Influence of scanner, powder application, and adjustments on
CAD-CAM crown misfit. J Prosthet Dent.

[36] Shim JS, Lee JS, Lee JY, Choi YJ, Shin SW, Ryu JJ (2015). Effect of software version and parameter settings on the marginal
and internal adaptation of crowns fabricated with the CAD/CAM
system. J Appl Oral Sci.

[37] Peroz I, Mitsas T, Erdelt K, Kopsahilis N (2019). Marginal adaptation of lithium disilicate ceramic crowns cemented
with three different resin cements. Clin Oral Investig.

[38] Zhang Y, Dudley J (2019). The influence of different cement spaces on the marginal gap of
CAD/CAM all-ceramic crowns. Aust Dent J.

[39] Zimmermann M, Valcanaia A, Neiva G, Mehl A, Fasbinder D (2018). Influence of Different CAM Strategies on the Fit of Partial Crown
Restorations: A Digital Three-dimensional Evaluation. Oper Dent.

[40] Saab RC, da Cunha LF, Gonzaga CC, Mushashe AM, Correr GM (2018). Micro-CT Analysis of Y-TZP Copings Made by Different CAD/CAM
Systems: Marginal and Internal Fit. Int J Dent.

[41] Borba M, Cesar PF, Griggs JA, Della Bona Á (2011). Adaptation of all-ceramic fixed partial dentures. Dent Mater.

[42] de Paula Silveira AC, Chaves SB, Hilgert LA, Ribeiro AP (2017). Marginal and internal fit of CAD-CAM-fabricated composite resin
and ceramic crowns scanned by 2 intraoral cameras. J Prosthet Dent.

